# Randomized trial of iReadMore word reading training and brain stimulation in central alexia

**DOI:** 10.1093/brain/awy138

**Published:** 2018-06-15

**Authors:** Zoe V J Woodhead, Sheila J Kerry, Oscar M Aguilar, Yean-Hoon Ong, John S Hogan, Katerina Pappa, Alex P Leff, Jennifer T Crinion

**Affiliations:** 1 Department of Brain Repair and Rehabilitation, Institute of Neurology, University College London, UK; 2 Department of Experimental Psychology, University of Oxford, UK; 3 Institute of Cognitive Neuroscience, University College London, UK; 4 Wellcome Trust Centre for Neuroimaging, University College London, UK; 5 Facultad de Psicología, Pontificia Universidad Javeriana, Colombia; 6 Experimental Psychology, University College London, UK

**Keywords:** central alexia, aphasia, computer-based rehabilitation, transcranial direct current stimulation (tDCS), reading therapy

## Abstract

Central alexia is an acquired reading disorder co-occurring with a generalized language deficit (aphasia). We tested the impact of a novel training app, ‘iReadMore’, and anodal transcranial direct current stimulation of the left inferior frontal gyrus, on word reading ability in central alexia. The trial was registered at www.clinicaltrials.gov (NCT02062619). Twenty-one chronic stroke patients with central alexia participated. A baseline-controlled, repeated-measures, crossover design was used. Participants completed two 4-week blocks of iReadMore training, one with anodal stimulation and one with sham stimulation (order counterbalanced between participants). Each block comprised 34 h of iReadMore training and 11 stimulation sessions. Outcome measures were assessed before, between and after the two blocks. The primary outcome measures were reading ability for trained and untrained words. Secondary outcome measures included semantic word matching, sentence reading, text reading and a self-report measure. iReadMore training resulted in an 8.7% improvement in reading accuracy for trained words (95% confidence interval 6.0 to 11.4; Cohen’s *d* = 1.38) but did not generalize to untrained words. Reaction times also improved. Reading accuracy gains were still significant (but reduced) 3 months after training cessation. Anodal transcranial direct current stimulation (compared to sham), delivered concurrently with iReadMore, resulted in a 2.6% (95% confidence interval −0.1 to 5.3; *d* = 0.41) facilitation for reading accuracy, both for trained and untrained words. iReadMore also improved performance on the semantic word-matching test. There was a non-significant trend towards improved self-reported reading ability. However, no significant changes were seen at the sentence or text reading level. In summary, iReadMore training in post-stroke central alexia improved reading ability for trained words, with good maintenance of the therapy effect. Anodal stimulation resulted in a small facilitation (*d* = 0.41) of learning and also generalized to untrained items.

## Teaser

Woodhead *et al.* present results from a randomized trial of a novel reading therapy app (‘iReadMore’) coupled with anodal transcranial direct current stimulation (tDCS) in patients with post-stroke central alexia. Use of iReadMore improves reading accuracy for trained words, while concurrent tDCS facilitates training and improves generalization to untrained stimuli.

## Introduction

Acquired disorders of reading may be a consequence of generalized language impairment. We refer to these disorders as central alexias (but see Ellis and Young, 1988; Warrington and Shallice, 1980, for a slightly different use of this term). Central alexia encompasses phonological, deep and surface alexia (Leff and Behrmann, 2008). Patients with central alexia are slow to read, make frequent errors and have additional problems with spoken language. We tested two concurrent therapies aiming to improve word reading in patients with central alexia after left hemisphere stroke: (i) ‘iReadMore’, a novel reading therapy app; and (ii) anodal transcranial direct current stimulation (tDCS) delivered to left inferior frontal gyrus (LIFG).

According to the primary systems hypothesis and connectionist triangle model of reading (Plaut *et al.*, 1996), central alexia may be due to damage to the phonological (P), semantic (S) or orthographic (O) system, or the connections between them (see Coltheart *et al.*, 2001; Coltheart, 2006, for a different theory of reading and the causes of phonological and surface dyslexia). Damage affecting phonology or the direct O-P mappings primarily impairs pseudoword reading (phonological alexia; Patterson and Lambon-Ralph, 1999; Crisp and Lambon Ralph, 2006) and causes semantic errors in more severe cases (deep alexia; Crisp *et al.*, 2011). Damage to the semantic system or the semantically (S) mediated O-S-P route impairs irregular word reading (surface alexia; Patterson and Lambon Ralph, 1999; Woollams *et al.*, 2007).

A number of therapies for central alexiahave been tested, mostly in single case experimental designs. Attempts to retrain grapheme-to-phoneme conversion rules or phonomotor processing have met with mixed success in phonological and deep alexia (De Partz, 1986; Mitchum and Berndt, 1991; Nickels, 1992; Conway *et al.*, 1998; Kendall *et al.*, 1998, 2003; Adair *et al.*, 2000; Kiran *et al.*, 2001; Friedman and Lott, 2002; Yampolsky and Waters, 2002; Stadie and Rilling, 2006; Kim and Beaudoin-Parsons, 2007; Brookshire *et al.*, 2014*a*; Riley and Thompson, 2014). Such sublexical approaches can be painstakingly slow, but have the advantage of generalizing to untrained words. Conversely, lexical approaches, e.g. crossmodal paired associate learning, priming or semantic remediation, have proven effective in phonological, deep and surface alexia, but tend not to generalize (Friedman and Robinson, 1991; Friedman *et al.*, 2002; Ska *et al.*, 2003; Kurland *et al.*, 2008; Lott *et al.*, 2008).

iReadMore uses a crossmodal, lexical approach, pairing written (O), spoken (P) and pictorial (S) representations of words over multiple trials with adaptive difficulty. It aims to strengthen connections between O, P and S domains, and hence has the potential to benefit all types of central alexia. We hypothesized that iReadMore would improve reading accuracy for trained words, but like other lexical therapies, would not generalize to untrained words.

iReadMore is based on a prototype reported by Woodhead *et al.* (2013). In that trial (in patients with pure alexia) functional imaging data indicated that training strengthened feedback connections from LIFG to visual cortex. Hence, we hypothesized that anodal tDCS delivered to LIFG during training may enhance feedback and facilitate therapy effects. This tDCS montage delivered concurrently with language therapy has been shown to improve speech production in chronic post-stroke aphasia (Baker *et al.*, 2010; Marangolo *et al.*, 2011, 2013; Campana *et al.*, 2015); reading in pure alexia (Lacey *et al.* 2015); and spelling in primary progressive aphasia (Tsapkini *et al.*, 2014). There have been no studies of tDCS in central alexia to date.

The effects of iReadMore and anodal tDCS were tested in a repeated-measures crossover design. Each patient received two 4-week blocks of iReadMore therapy, accompanied with either real (anodal) or sham tDCS. Change in reading ability for trained and untrained words after iReadMore training was assessed, and compared for real versus sham stimulation. A subset of the 50 most frequent English words (‘core’ words), mostly low imageability function words, were trained in both blocks due to their importance for reading, and to test the hypothesis that words with low semantic content could also be trained using iReadMore.

## Materials and methods

### Study design

**Figure 1:**
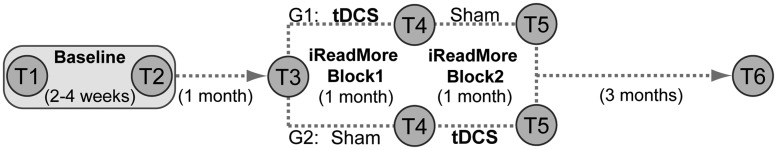
**Study design.** G1 = Group1: received tDCS in Block 1 and sham in Block 2. G2 = Group2: received sham in Block 1 and tDCS in Block 2.

A repeated-measures crossover design with six time points (T1–T6) was used (Fig. 1). T1–T5 were spaced by 4-week intervals. Baseline language tests were spread over T1 and T2 and combined. The interval between T2 and T3 was used to assess pre-therapy (test-retest) changes. Two 4-week therapy blocks followed: Block 1 from T3 to T4 and Block 2 from T4 to T5. T6 measured therapy maintenance 3 months after completion of training.

During therapy blocks participants attended three 40-min face-to-face sessions per week (Monday, Wednesday and Friday), where iReadMore was administered concurrently with anodal tDCS or sham tDCS. Participants completed additional behavioural training using iReadMore independently at home to amass at least 35 h total practice per block.

Half the participants (Group 1) received anodal tDCS in Block 1 and sham in Block 2. The other half (Group 2) received sham then anodal tDCS. Block randomization with bias minimization was used to allocate participants to Group 1 or Group 2 and ensure crossover groups did not become unbalanced on severity (baseline word reading accuracy and speed). Numerical codes for anodal tDCS and sham conditions were prepared independently in advance of the trial (J.C.) and executed by the researchers (S.K., Z.W.). Participants and researchers collecting and analysing the data were blinded to tDCS condition using the stimulator’s study mode. Unblinding occurred after data acquisition and analysis ended.

Control participants took part in one testing session to provide normative data on word and pseudoword reading tests (Table 1).

**Table 1:** Demographic characteristics and baseline assessment scores for 21 participants with central alexia, ordered by tDCS group and word reading accuracy

ID	Sex	Age, years	Time since stroke, months	Lesion volume, cm^3^	CAT naming, %^*^	Word reading, %	Pseudo. Reading, %	CA type
**tDCS Group 1**							
P7	M	67	107	12	72	12	3	D
P18	M	72	101	243	9	13	0	D
P16	M	60	16	103	33	28	10	D
P2	M	50	82	305	53	40	0	D
P1	M	44	94	241	69	58	0	D
P8	F	43	55	399	81	58	20	D
P13	M	56	23	45	72	80	0	P
P5	F	55	75	151	93^a^	92	30	P
P12	M	54	24	149	86	92	65	P
P10	M	52	12	34	88	96	75^a^	P
**tDCS Group 2**							
P9	M	61	19	196	40	3	0	D
P15	M	73	158	205	71	20	0	D
P17	F	50	72	141	28	36	5	P
P14	M	54	39	190	14	47	3	P
P19	F	58	41	298	81	59	0	P
P4	F	56	93	150	5	64	75^a^	S
P3	M	52	66	123	66	71	0	P
P20	M	42	13	44	72	75	28	P
P21	F	26	81	162	79	76	0	D
P6	F	33	59	181	95^a^	90	3	P
P11	F	50	14	59	83	91	25	P
**Patient mean (SD)**		53 (11)	59 (39)	163 (99)	61 (28)	57 (30)	16 (25)	–
**Patient range**		26–73	12–158	12–399	5–95	3–96	0–75	–
**tDCS Group 1 Mean (SD)**		55 (9)	59 (36)	168 (121)	66 (25)	57 (31)	20 (27)	–
**tDCS Group 2 mean (SD)**		50 (13)	60 (41)	159 (66)	55 (29)	57 (27)	13 (22)	–
**Control mean (SD)**		53 (12)	–	–	–	100 (1)	93 (11)	–
**Control range**		23–70	–	–	–	98–100	50–100	–

Type of central alexia (CA) diagnosed is also presented: phonological alexia (P), deep alexia (D) or surface alexia (S).

*CAT naming aphasia cut-off <91.6%.

^a^Denotes patient scores that were not significantly impaired relative to the control data or CAT naming cut-offs.

Testing and face-to-face therapy sessions were conducted at the Institute of Cognitive Neuroscience, University College London.

### Participants

**Figure 2:**
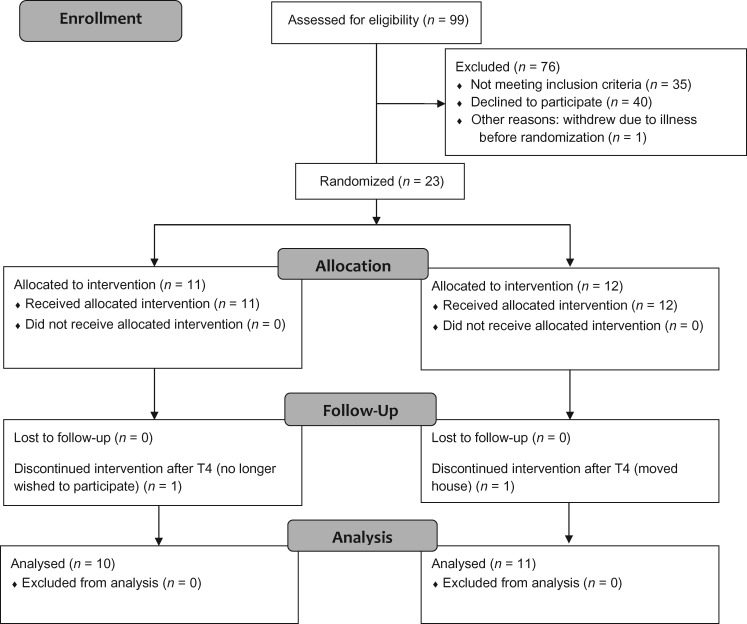
**Consolidated Standards of Reporting Trials (CONSORT) flow diagram.**.

Data are presented from 21 patients (13 male; Table 1 and Fig. 2) with a clinical diagnosis of post-stroke aphasia (made by either a neurologist or a speech and language therapist). All patients suffered a left-sided, middle cerebral artery territory stroke and had a reading disorder caused by their generalized language impairment. They were recruited between January 2014 and March 2016 mainly from the PLORAS database (Wellcome Trust Centre for Neuroimaging, UCL; Seghier *et al.*, 2016), but also from a local specialist aphasia service. Twenty participants exhibited phonological (*n* = 11) or deep (*n* = 9) alexia and one exhibited surface alexia (Patient P4). Details on the methods categorizing patients to each group, based on Whitworth *et al.* (2014), can be found in the Supplementary material. This incidence ratio is consistent with a study of 69 stroke patients with central alexia (Brookshire *et al.*, 2014*b*); surface alexia is more commonly encountered in patients with semantic dementia (Woollams *et al.*, 2007).

Sample size was calculated using alpha = 5% and beta = 90% (see Supplementary material for more details). Results using a prototype of iReadMore (Woodhead *et al.*, 2013) indicated 18 subjects would be required to detect a comparable change. LIFG anodal tDCS results reported in a study by Baker and colleagues (2010) indicated 13 subjects would be required. Taking a conservative approach to allow for possible differences between studies, we aimed to collect data from 20 subjects. Recruitment stopped at *n* = 24 (to allow for a 20% drop-out rate), of whom *n* = 21 participants completed the protocol (Fig. 2).

Inclusion criteria included: (i) left-hemisphere middle cerebral artery stroke; (ii) more than 1-year post-stroke; (iii) dominant English language use in activities of daily living; and (iv) central alexia, operationalized as impaired word reading [Comprehensive Aphasia Test (CAT; Swinburn *et al.*, 2005) word reading T-score < 61] and impaired spoken language (CAT naming < 63 or picture description < 61).

Exclusion criteria included: (i) premorbid history of neurological or psychiatric illness; (ii) history of developmental language disorder; (iii) severe spoken output deficit and/or speech apraxia (CAT repetition < 44); (iv) seizures in the past 12 months; (v) contraindications to MRI scanning; and (vi) extensive damage to LIFG.

Group 1 comprised 10 participants (alexia subtypes: seven phonological, three deep, one surface) and Group 2 comprised 11 participants (alexia subtypes: four phonological, six deep). There were no between-group differences in age, time since stroke, lesion volume, training dose or baseline word reading performance (independent-samples *t*-tests, *P* > 0.3 in all cases); the number of male and female participants (Fisher’s exact test, *P* = 0.18); or the number of participants showing a phonological or deep alexia subtype (Fisher’s exact test, *P* = 0.37).

Control data for word and pseudoword reading tests (Table 1) were collected from 21 age- and sex-matched healthy participants. There was no significant difference in age between patient and control groups (*P* = 0.84). Control participants spoke English as their dominant language, and had no history of neurological or psychiatric illness or developmental language disorder.

All participants gave written informed consent. The protocol was approved by the London Queen Square Research Ethics Committee, UCL.

### Structural MRI acquisition and lesion identification

Structural whole brain MRI data were acquired for lesion identification using a multi-parameter mapping protocol with a 3.0 T whole body magnetic resonance system (Magnetom TIM Trio, Siemens Healthcare) and a 32-channel transmitter-receiver head coil. We used quantitative magnetization transfer (MT) maps from a multi-parameter mapping protocol described by Callaghan *et al.* (2015) due to their excellent contrast and spatial resolution.

MT maps were created using the Voxel Based Quantification toolbox in SPM12 (http://www.fil.ion.ucl.ac.uk/Research/physics_info/QuantMRI_VBM.html; http://www.fil.ion.ucl.ac.uk/spm/). The ALI toolbox (Seghier *et al.*, 2008) was used for MT map normalization, segmentation and lesion identification.

### Training and testing word lists

Words with high written frequency (SUBTLEX_WF_ > 50) were selected from the SUBTLEX database (Brysbaert and New, 2009). High frequency words were chosen to maximize the ecological utility of the therapy. All words were three to six letters long so that they could easily be read in one fixation. Hyphenated or punctuated words were excluded, and an effort was made to avoid regular morphological variants of the same word (e.g. eat, eaten, eating). Words of all classes (nouns, verbs, functors etc.) were included, including words that have either high or low imageability ratings. See Supplementary Fig. 1 for details of the training and testing word lists.

Three matched lists of 180 words were created (A, B and C). For each word on List A there was a corresponding word on Lists B and C closely matched for letter length, syllable length, written frequency and imageability. Additionally, the 50 highest frequency words (mostly function words) were selected as a separate list of ‘core’ words.

All 590 words were tested at baseline (split across T1 and T2 sessions). Results from this full corpus of testing items were used to establish the participants’ profiles of reading impairment (Supplementary Table 2). Based on each participant’s baseline performance, a customized set of 150 matched words from each of the A, B and C lists were selected to use in training and subsequent assessments. This ensured the A, B and C lists selected for that patient were matched for baseline reading performance (word reading accuracy and reaction time). Furthermore, the lists remained matched for psycholinguistic variables. The A, B and C word lists were assigned to be either trained in Block 1, trained in Block 2 or not to be trained (untrained words). List allocations were counterbalanced between participants. All 50 core words were trained in both Block 1 and Block 2 because of their high utility.

From the customized 150-item A, B and C word lists, a subset of 90 items from each list were selected for use in all subsequent assessment time points (T3–T6). These 90-item testing lists were matched for baseline performance and psycholinguistic variables. Importantly, the overall accuracy of the word lists selected for testing was matched to baseline reading accuracy to avoid the risk of regression to the mean at future time points. A subset of 30 core words were tested at T3–T6. Hence, 300 words were tested at T3–T6 sessions. Results from this subset of testing items was used to report the change in reading performance from T1 to T6 (Fig. 4).

### iReadMore training

Training was delivered using iReadMore on a tablet computer, which automatically recorded training duration.

The software cycled through ‘exposure’ and ‘challenge’ phases. During exposure phases, participants passively viewed 10 trials wherein a picture, symbol or visual mnemonic representing the target word was presented, followed by simultaneous presentations of the written and spoken word-forms. Written word duration initially matched the patient’s baseline word reading speed, then adapted according to performance in the subsequent challenge phase. Stimuli recordings from a female or a male speaker were randomly selected for each trial.

Challenge phases comprised up to 30 trials. In each trial, a spoken word from the preceding exposure phase was presented with a written word. In half the trials the written and spoken stimuli were the same word, and in half they were different. Participants made a same/different response via button press and received immediate feedback. Two points were awarded for a fast correct response; 1 point for a slow correct response; and −1 for an incorrect response. If the participant reached the criterion score within 30 trials, they passed that level and task difficulty increased in the next exposure phase.

Task difficulty was reflected in three adaptive parameters: (i) written word duration in exposure and challenge phases; (ii) criterion score in the challenge phase; and (iii) criterion duration for fast/slow correct responses in the challenge phase. All three parameters increased incrementally when the participant passed a level. If the participant failed three successive levels, the parameters reverted back by one increment.

Same/different task difficulty was adapted independently on a word-by-word basis. Each target word (e.g. ‘hand’) was paired with easy, medium or hard distractors varying in the number of letters shared with the target word (e.g. ‘heap’, ‘hood’, or ‘hard’). All distractors shared the same length and first letter with the target word. The distractor selected for each trial started at the easy level and increased or decreased according to response accuracy.

Further details on the iReadMore training procedure are available in the Supplementary material.

### Transcranial direct current stimulation

Stimulation was delivered concurrently with iReadMore for the first 20 min of each face-to-face session using a NeuroConn battery powered constant current stimulator (http://www.neuroconn.de/dc-stimulator_plus_en/).

Two 35 cm^2^ rubber electrodes in saline soaked sponges were secured with elastic straps and self-adhesive bandages. The anode was positioned over left frontal cortex (10-10 position FC5) and the cathode over right supraorbital region.

Stimulation duration and intensity complied with current safety recommendations (Fregni *et al.*, 2015). Anodal tDCS used 15 s fade-in, 20 min continuous stimulation at 2 mA, and 15 s fade-out. The active sham stimulation used 15 s fade-in, 30 s 2 mA direct current, 15 s fade-out and 20 min without any stimulation, but with continuous impedance control. This does not affect neural functions, but assures effective blinding of participants due to the initial tingling sensation on the scalp (Gandiga *et al.*, 2006).

Before and after tDCS participants rated how comfortable they felt from 1 (very uncomfortable) to 10 (very comfortable). Patients were asked to report any adverse events experienced during or between stimulation sessions.

### Baseline behavioural assessment

At baseline (T1–T2 testing sessions), patients were tested with custom-made word reading and pseudoword reading tests, and the picture naming test from the CAT. Controls provided normative word and pseudoword reading data, but control naming data were not acquired as published norms are available. Each patient’s scores were compared to control data using the Crawford Singlims program (http://homepages.abdn.ac.uk/j.crawford/pages/dept/psychom.htm#conflims; Crawford and Garthwaite, 2002) to establish the profile of impairment.

Baseline word reading ability was assessed in patients using the full corpus of 590 words (540 A/B/C words plus 50 core words) tested over T1 and T2 baseline testing sessions. Words were presented in a random order and split into six separated blocks, three at each testing session. Controls were assessed on a representative sample of 127 words tested in one block.

Words were presented for up to 4 s in black, lowercase, size 36 pt Arial font on a grey background using E-prime (Schneider *et al.*, 2012). Participants were instructed to read the words aloud into a voice-key microphone as quickly and accurately as they could. Accuracy was recorded online by experimenter button press. One point was awarded for correct responses; 0.5 for self-corrections; and 0 for incorrect responses. Reading reaction time was recorded by the voice key. Reaction times were excluded for incorrect or self-corrected trials, voice-key failure and trial with reaction time >2 standard deviations (SD) from the mean. Patients were classified into alexia subtypes (phonological, deep or surface alexia) based on their word reading errors and any imageability, regularity and lexicality effects shown in their accuracy data from the baseline word reading and pseudoword reading assessments. For more details, see Supplementary Table 2.

Pseudoword reading ability was assessed in patients and controls using 20 pseudowords, three to six letters in length, with plausible letter combinations generated using Wuggy software (Keuleers and Brysbaert, 2010). Pseudowords were presented and scored in the same manner as real words but without the four seconds timeout.

### Interval behavioural assessments

Outcome measures were assessed once at baseline (T1 or T2) and at every subsequent time point (T3–T6).

#### Primary outcome: Word Reading Test

The primary outcome measures were word reading accuracy and reaction time (calculated using correct trials only, and excluding trials where the reaction time was >2 SD from the subject’s mean). After baseline, a personalized subset of 90 words from each word list (A–C) and 30 items from the core word list were selected for each participant and tested at all subsequent time points. A–C items were matched for baseline reading performance. Words were presented in a randomized order across three runs.

#### Secondary outcomes

##### Written semantic matching

This task assessed silent reading for meaning. In each trial (presented in E-Prime), participants silently read three words: a probe word at the top of the screen, a semantically-related target and an unrelated distractor below. Participants were instructed to identify the target as quickly as possible by button press. Percentage accuracy and mean reaction time (for correct trials only, excluding trials where reaction time >2 SD from the mean) were calculated.

The three words for each trial were drawn from the same word list (A, B or C). Twenty-four trials for each list were presented in a randomized order. The stimuli for each list were matched for number of letters, frequency, imageability and regularity.

##### Sentence reading

This task assessed silent sentence reading. In each trial (presented in E-prime), participants silently read a sentence of five to eight words as quickly as possible, then pressed a button when finished. This response was used to calculate reading speed in words per minute (excluding trials with reaction time >2 SD from the mean). Next, a picture was displayed and the participant responded verbally whether the picture was congruent with the sentence or not. Percentage accuracy on the picture verification task was calculated.

Ten sentences for each word list were created, each containing between two to four words from the list. For example, the sentence ‘He *sold* the *broken camera*’ contained the words ‘sold’, ‘broken’ and ‘camera’ from List A. The sentences from each word list were matched for sentence structure, number of trained words, total number of words, and summed word imageability, regularity, frequency and letter length.

##### Text reading

Text reading was assessed using passages one and two from the Neale Analysis of Reading Ability (Neale, 1999). The two forms of the test were counterbalanced across participants and between time points. Reading accuracy was recorded for each word. If a participant could not read a word within 4 s, the experimenter supplied the word and it was scored as incorrect. Outcome measures were percentage of words read correctly (accuracy), reading speed in words per minute and total score on the subsequent comprehension questions.

##### Sustained Attention to Response Task

A non-verbal version of the Sustained Attention to Response Task (SART) (Robertson *et al.*, 1997) assessed domain-general changes resulting from iReadMore or tDCS. The Go/No-Go task contained pictures of two different people, one of which was revealed in each trial. Participants were instructed to press a button whenever one identified person appeared (Go trial), but withhold their response for the other (No-Go trial). One hundred and ninety-two Go trials and 24 No-Go trials were presented in a pseudorandomized order. Outcome measures were the number of false negative and false positive responses and the mean reaction times on correct Go trials.

##### Self-report measures

The reading subtest of the Communication Disability Profile (CDP) (Swinburn and Byng, 2006) was administered at T3 and T5 to investigate change in the participants’ self-reported reading ability. Using a scale from bad (0) to good (4) they rated their silent reading ability over the past week at the level of: single words; sentences; texts; and written correspondence, i.e. letters. The outcome measure was their total score out of 16.

Upon completion of the therapy (T5), participants completed an exit questionnaire where they judged whether their word reading had improved (No / A little / A lot); whether they wished to continue using iReadMore; and whether they had noticed any difference in stimulation effects in Blocks 1 and 2.

### Planned analyses

Planned analyses were conducted as stated in the clinical trials registration (www.clinicaltrials.gov, NCT02062619).

For each outcome measure, an ‘Omnibus’ analysis was applied to investigate overall changes in performance across all time-points. A more focused ‘Therapy’ analysis investigated immediate therapy effects of iReadMore and anodal tDCS in Blocks 1 and 2.

Where multiple variables were produced from a single test (e.g. accuracy and reaction time measures from the Word Reading Test), the Omnibus analysis used a multivariate ANOVA (MANOVA). If not, a univariate ANOVA was used.

The Omnibus (M)ANOVA had the following factors: within-subjects effect of time point (Baseline, T3, T4, T5 and T6); within-subjects effect of word list (where appropriate: Trained in Block 1, Trained in Block 2 and Untrained); and between-subjects effect of tDCS Group [Group 1 (anodal tDCS in Block 1), Group 2 (anodal tDCS in Block 2)].

The Therapy analysis used a repeated-measures ANOVA with factors: within-subjects effect of Block [change in Block 1 (T3–T4), change in Block 2 (T4–T5). Change was simply calculated as the difference from one time point to the other]; within-subjects effect of word list; between-subjects effect of tDCS Group

For the CDP (administered at T3 and T5), scores were compared using Wilcoxon Signed Rank tests.

Cohen’s *d* effect sizes were calculated for changes in the primary outcome measure, word reading accuracy and reaction time.

### Exploratory analyses

*Post hoc* exploratory analyses were conducted to explore additional aspects of the results.

We tested whether changes in word reading ability during Blocks 1 and 2 were larger than the test-retest effects between baseline and T3. This was done using paired *t*-tests comparing change in trained word reading accuracy and reaction time over Blocks 1 and 2 to changes in the same measures between baseline and T3.

Maintenance of therapy effects on word reading ability were assessed with paired *t*-tests comparing scores immediately before treatment (T3) to the follow-up testing session at T6.

We tested whether word imageability or regularity influenced the efficacy of reading therapy. To do this, the full word corpus (180 words in three different lists) was ranked in order of imageability. Words in the lowest 40th percentile were labelled as low imageability; words in the highest 60th percentile were labelled as high imageability. For the regularity analysis, words were classified as either regular or irregular. We then calculated each subject’s improvements in trained word reading over Blocks 1 and 2 for words with high/low imageability, and for regular/irregular words. The results were then averaged over the two blocks. Finally, four paired *t*-tests were computed, testing the effect of word imageability and regularity on change in trained word reading accuracy and reaction time.

## Results

### Lesion overlay mapping

**Figure 3:**
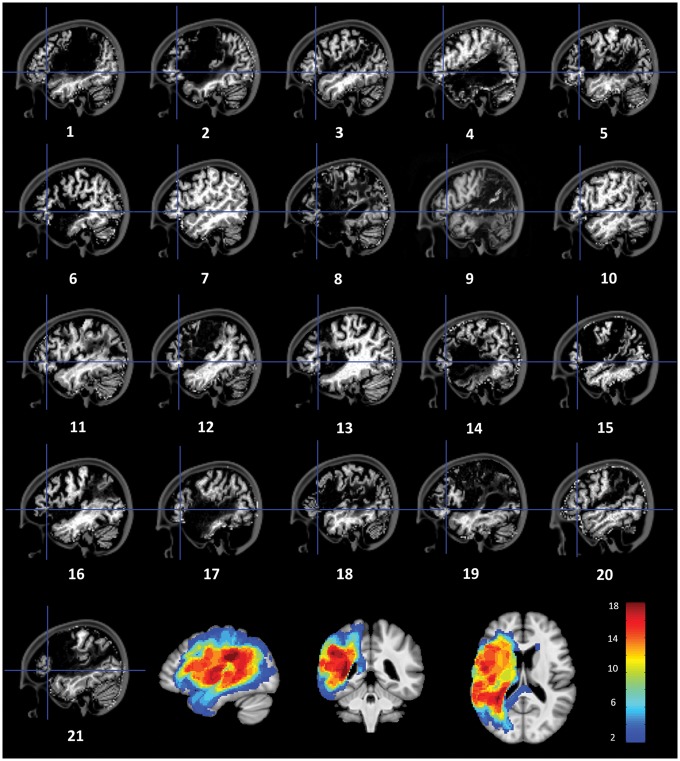
**Patient structural MRI images and lesion overlap map.** Crosshairs indicate the approximate location of the stimulation site. *Bottom right* tiles show the lesion overlay map with voxels where at least two patients had damage. The highest lesion overlap (*n* = 20) was seen in two areas: (i) the superior longitudinal fasciculus underlying the supramarginal gyrus; and (ii) the junction of the superior longitudinal, inferior longitudinal and inferior fronto-occipital fasciculi underlying the posterior superior temporal sulcus.

The lesion overlay map (Fig. 3) showed group damage throughout left perisylvian middle cerebral artery territory. All patients had some anatomically spared tissue in LIFG. Adjacent pars opercularis and/or premotor cortex were damaged in 14 patients.

### Baseline reading and naming impairments

The results of the baseline word reading, pseudoword reading and CAT naming tests are presented in Table 1. Crawford’s test showed that all patients were significantly impaired on word reading accuracy, and all except Patient P20 were significantly impaired on word reading reaction time. All participants except Patients P04 and P10 showed significantly impaired pseudoword reading accuracy. Finally, all patients except Patients P5 and P6 showed CAT naming abilities below the aphasia cut-off criterion.

### iReadMore training dose

On average, patients completed 34.6 h of iReadMore training in Block 1 (range: 29.9–37.4) and 35.2 h in Block 2 (range: 30.0–41.4). At the within-subjects level there was little change in dose for Blocks 1 and 2; the difference was 80 min on average and the maximum difference was 6 h 16 min (Patient P4). A repeated-measures ANOVA on iReadMore dose showed no significant effects of Block, tDCS Group or Interaction (*P* > 0.1 in all cases).

Information on participant performance on the same/different challenge phase task is available in the Supplementary material.

### Transcranial direct current stimulation adverse events

Patients reported only mild adverse events, including fatigue, headaches and skin irritation. No adverse event was severe enough to warrant cessation of stimulation. Adverse event frequency did not differ during anodal tDCS versus sham [*t*(20) = 2.3, *P* = 0.82].

The effect of stimulation on comfort ratings was calculated as rating before stimulation minus rating after stimulation, with a maximum possible change of 10. The average change was small: −0.05 for anodal tDCS (range: −0.8 to +0.9) and −0.18 for sham (−1.47 to 0.45). There was no significant difference between anodal tDCS and sham blocks [*t*(20) = 1.6, *P* = 0.12].

In the exit questionnaire 10/21 participants said stimulation felt different in the two blocks. Of those, 6/10 commented on which block contained real tDCS stimulation: unblinding revealed that 4/6 were correct. All participants reported that they found tDCS tolerable and would be willing to continue receiving it if it were available in future.

### Behavioural effects of therapy

Average outcome measures for both tDCS Groups and results from the Omnibus and Therapy (M)ANOVAs are reported in Supplementary Table 1.

### Primary outcomes

#### Word reading accuracy

**Figure 4:**
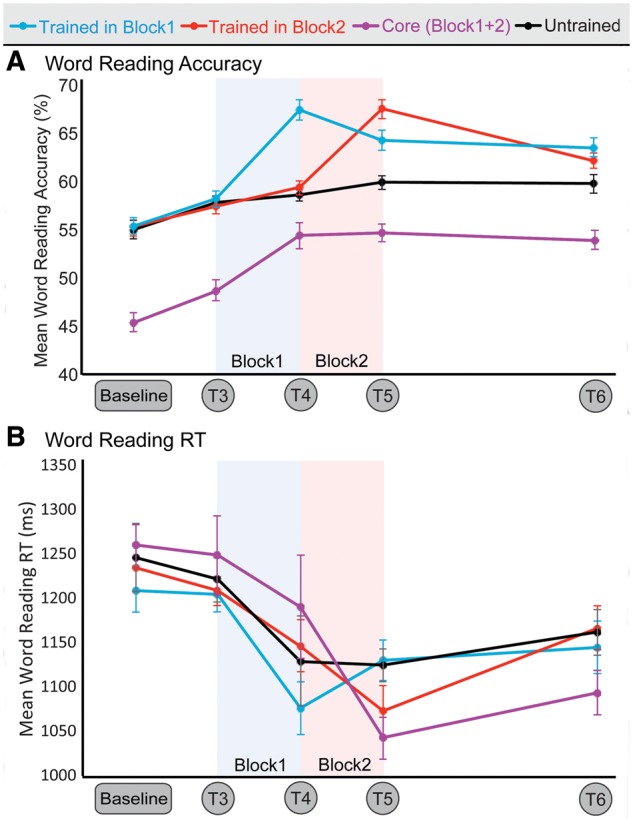
**Therapy effects on word reading ability.** Change over time in (**A**) mean word reading accuracy (*n* = 21) and (**B**) reaction times (*n* = 20). There were four different word lists: words trained in Block 1 (blue), words trained in Block 2 (red), untrained words (black) and the unmatched list of high-frequency, low-imageability core words (purple). Error bars indicate within-subject standard error of the mean (SEM). Training Block 1 was administered between T3 and T4; Block 2 was administered between T4 and T5.

Overall change in word reading accuracy is shown in Fig. 4A. All word lists showed a test-retest effect between baseline and T3. Between T3, T4 and T5, therapy effects specific to trained words were observed. Between T5 and the follow-up test at T6 reading ability diminished, but stayed above baseline levels.

The item-specific therapy effects of iReadMore training on word reading accuracy were observed in the Therapy ANOVAs as a significant Block × Word list interaction (*P < *0.0005). Unstandardized and standardized effect sizes for changes in word reading accuracy are shown in Table 2. Combining data from both blocks, the average improvement in trained word reading accuracy was 8.7% [95% confidence interval (CI) 6.0–11.4; *d* = 1.38]. Exploratory *post hoc* paired *t*-tests showed that the improvement in trained word reading accuracy (during Blocks 1 and 2) was significantly greater than the test-retest effects observed between Baseline and T3 [Block 1: *t*(20) = 3.3, *P < *0.005; Block 2: *t*(20) = 3.5, *P < *0.005].

**Table 2:** Unstandardized effect sizes (with 95% CI) and standardized effect sizes (Cohen’s *d*) for changes in the primary word reading outcome measures

**Measure**	**Time interval**	**Unstandardized effect size(95% CI)**	**Cohen’s *d***
**Word reading, accuracy %**			
Trained in Block 1	T4 − T3	9.2 (6.2 to 12.3)	1.29
Untrained	T4 − T3	0.7 (−1.3 to 2.7)	0.16
Trained in Block 2	T5 − T4	8.1 (5.3 to 10.9)	1.25
Untrained	T5 − T4	1.3 (−0.6 to 3.1)	0.29
Trained, both blocks	After − Before	8.7 (6.0 to 11.4)	1.38
**Word reading, reaction time, ms**			
Trained in Block 1	T4 − T3	−128 (−53 to −202)	0.75
Untrained	T4 − T3	−92 (44 to −228)	0.30
Trained in Block 2	T5 − T4	−73 (−4 to −142)	0.47
Untrained	T5 − T4	−4 (117 to −125)	0.01
Trained, both blocks	After − Before	−100 (−56 to −145)	0.98
**Core word reading, accuracy, %**	T4 − T3	5.7 (1.5 to 9.9)	0.58
	T5 − T4	0.3 (−2.4 to 3.0)	0.04
	T5 − T3	6.0 (2.7 to 9.2)	0.78
**Core word reading, reaction time, ms**	T4 − T3	−66 (−113 to −245)	0.17
	T5 − T4	−144 (−6 to −281)	0.47
	T5 − T3	−210 (−116 to −304)	1.00

**Figure 5:**
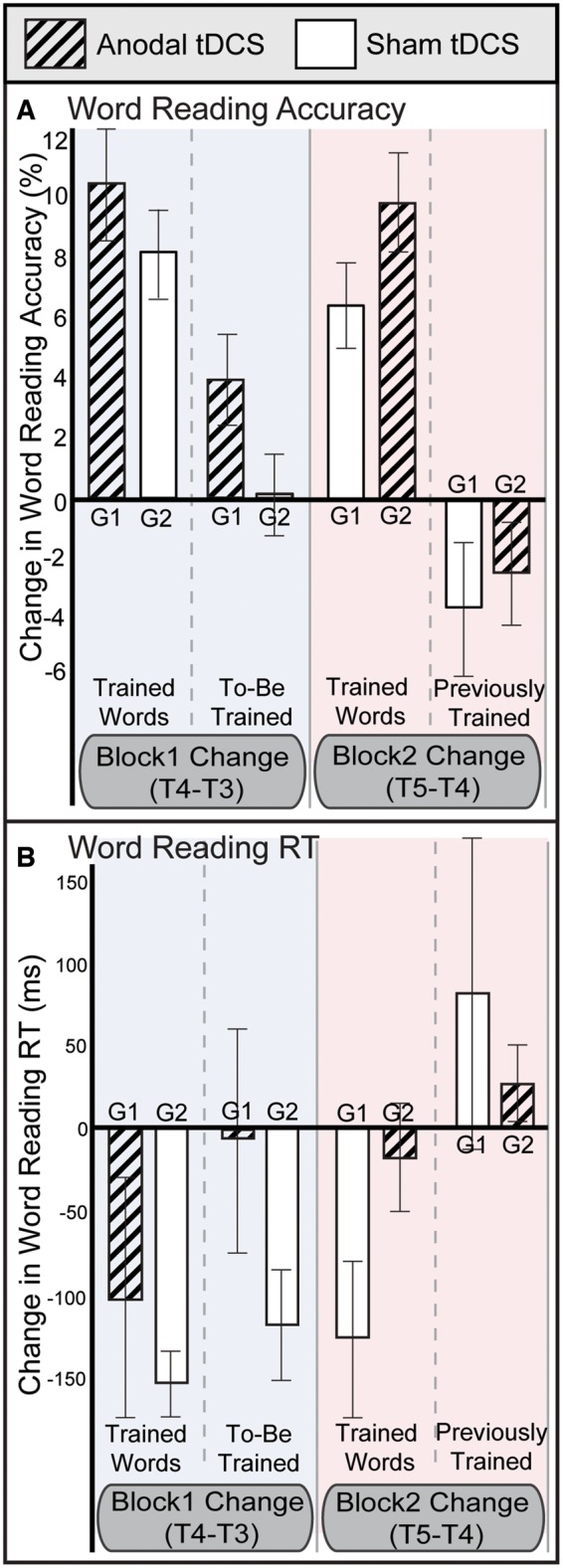
**Change in word reading ability after therapy.** Effects of iReadMore and tDCS on change in (**A**) word reading accuracy (*n* = 21) and (**B**) word reading reaction times (RT) (*n* = 20). Block 1 change was calculated as accuracy or reaction time at T4 − T3; Block 2 change was T5 − T4. G1 = cross-over group 1; G2 = cross-over group 2. Error bars represent the within-subject SEM.

As shown in Fig. 5, anodal tDCS also had a beneficial effect on word reading accuracy (Block × tDCS interaction, *P < *0.05), an effect which generalized to untrained words. Collapsing data from both word lists and blocks, accuracy improved by 2.6% more during anodal tDCS than sham (95% CI − 0.1 to 5.3; *d* = 0.41).

Maintenance of the iReadMore training effects were tested using *post hoc* paired *t*-tests to compare accuracy at T3 (immediately before training) and T6 (3 months after training cessation). Accuracy for all trained words was significantly better at T6 than T3 [Trained in Block 1: *t*(20) = 3.6, *P < *0.005; Trained in Block 2: *t*(20) = 3.9, *P < *0.005]. The improvement in untrained items was not significant [*t*(20) = 1.7, *P* = 0.10]. At T6, accuracy for trained words was significantly greater than for untrained words [Trained in Block 1: *t*(20) = 2.3, *P < *0.05; Trained in Block 2: *t*(20) = 3.3, *P < *0.005].

Maintenance of the tDCS effects were harder to assess because of the cross-over design, but reading accuracy at T6 was assessed with an ANOVA with within-subjects factor word list (Trained in Block 1 versus Trained in Block 2) and between-subjects factor tDCS Group; if the facilitatory effects of tDCS had persisted until T6, the interaction between word list and group should be significant. The interaction was not significant [*F*(1,19) = 0.4, *P* = 0.55].

Exploratory *post hoc* paired *t*-tests tested the hypothesis that the therapy may have been more effective for more imageable or more regular words. Neither factor had a significant effect on change in trained word reading accuracy [imageability: *t*(20) = 1.84, *P* = 0.081; regularity: *t*(20 = 1.18, *P* = 0.251]; in fact, for imageability, there was a trend for larger improvements for low imageability words (mean improvement = 9.76%, SD = 10.85) than high imageability words (mean improvement = 5.07%, SD = 5.90).

**Figure 6:**
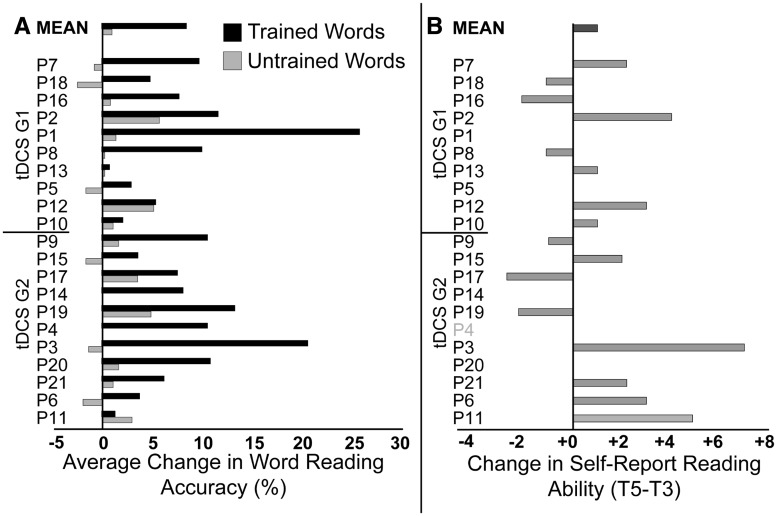
**Change in word reading accuracy and self-reported reading by participant.** (**A**) Raw percentage change in word reading accuracy for trained (black) and untrained (grey) words, averaged over Block 1 (T4 − T3) and Block 2 (T5 − T4). For trained words, this represents the average of the change in the 90 words trained in Block 1 between T3 and T4 and the change in the 90 (different) words trained in Block 2 between T4 and T5. For untrained words, this represents the change in the 90 untrained words over the same two time-periods. Participants are ordered according to tDCS group, followed by ascending CAT naming accuracy. (**B**) The CDP measures self-report ability in silent word, sentence, text and mail reading. Score for each level is out of 4, giving a total score out of 16. Change in CDP score is the difference between T5 (after training) minus T3 (before training). Positive scores represent improvements in self-reported reading ability. CDP data were unavailable for Patient P4.

At the individual subject level, there was considerable heterogeneity between participants. Figure 6 shows the change in word reading accuracy for trained and untrained words, averaged over both blocks, for each participant. This represents the average change over the 90 words trained in Block 1 and the 90 words trained in Block 2, compared to the change in the 90 untrained words across the same time-frame. More detailed plots showing the change over time, for each word list, and for each subject can be seen in Supplementary Fig. 3. The cause of this heterogeneity, which has considerable clinical relevance, is the subject of a parallel analysis currently being prepared for publication.

#### Word reading reaction times

Because of Patient P9’s low word reading accuracy, reaction time could not be calculated; hence reaction time data were available for 20 participants only. Overall change in word reading reaction time, shown in Fig. 4B, largely mirrored that of word reading accuracy: there was no indication of a speed-accuracy trade-off. A small test-retest effect was apparent between Baseline and T3. Between T3, T4 and T5, improvements were observed that were strongest for trained words. Between T5 and the follow-up test at T6 reading ability diminished, but stayed above baseline levels.

There was an item-specific therapy effect of iReadMore training on word reading reaction time, demonstrated by a significant Block × Word list interaction (*P < *0.05). Averaging across both blocks, the average unstandardized effect size of the improvement was 100 ms (95% CI 56 to 145; *d* = 0.98). *Post hoc* paired *t*-tests showed that the improvements in trained word reaction time were significantly greater than the test-retest effects (Baseline to T3) for Block 1 but not for Block 2 [Block 1: *t*(19) = 2.4, *P < *0.05; Block 2: *t*(19) = 1.2, *P* = 0.3].

The effect of tDCS on word reading reaction time was not significant.

Exploratory paired *t*-tests of maintenance effects compared word reading reaction time at T3 versus T6, and demonstrated that improvements in reaction time were not maintained at the follow-up session [Trained in Block 1: *t*(19) = 1.8, *P* = 0.09; Trained in Block 2: *t*(19) = 0.9, *P* = 0.36]. Similarly, at T6, there was no significant difference in reaction time between trained and untrained words [Trained in Block 1: *t*(19) = − 0.4*, P* = 0.67; Trained in Block 2: Trained in Block 2: *t*(19) = 0.3, *P* = 0.77].

*Post hoc* paired *t*-tests showed no significant effects of word imageability or regularity on improvement in word reading reaction time after iReadMore training [imageability: *t*(18) = − 1.18, *P* = 0.253; regularity: *t*(18) = 0.51, *P* = 0.62].

#### Core word reading accuracy

The core word list was analysed separately because it was trained in both blocks and items were not matched in psycholinguistic properties to the other lists. Core word reading accuracy improved in Block 1, but gains did not continue in Block 2.

*Post hoc* contrasts in the univariate Therapy ANOVA confirmed a significant improvement in accuracy between T3 and T4 [*F*(1,16) = 8.8, *P < *0.01]. In addition, *post hoc* paired subjects *t-*tests demonstrated that accuracy was better at T5 than T3 [*t*(20) = 3.6, *P < *0.005]. The unstandardized effect size for core word reading improvement between T3 and T5 was 6.0% (CI 2.7% to 9.2%), and the standardized effect size was *d* = 0.78. However, this change was not significantly larger than the test-retest effect observed between Baseline and T3 [*t*(20) = 1.0, *P* = 0.3].

There was no significant effect of tDCS for core word reading accuracy.

*Post hoc* paired *t*-tests comparing Core word reading accuracy at T3 versus T6 showed that improvements were maintained at the follow-up session [*t*(20) = 3.5, *P < *0.005].

#### Core word reading reaction times

As Patients P9 and P15 had very low core word reading accuracy, reaction time could only be calculated for 19 participants. In contrast to accuracy, core word reading reaction time improved marginally in Block 1 and more substantially in Block 2. *Post hoc* contrasts in the Therapy ANOVA confirmed that the change in reaction time between T4 and T5 was significant [*F*(1,16) = 4.7, *P < *0.05]. A paired *t*-test showed that the overall change between T3 and T5 (mean = 210 ms; 95% CI 116 to 304; *d* = 1.00] was significant [*t*(18) = 3.6, *P < *0.005]. This change was also significantly larger than the test-retest effect observed between Baseline and T3 [*t*(17) = 2.2, *P* < 0.05].

There was no significant effect of tDCS for core word reading reaction time.

Finally, a paired *t*-test comparing core word reading reaction time at T3 versus T6 showed a significant maintenance of therapy effects [*t*(18) = 2.5, *P* < 0.05].

### Secondary outcomes

#### Written semantic matching

Patients P7 and P9 were unable to complete the written semantic matching task due to their extremely poor word reading abilities. Data are reported from the remaining 19 participants.

Accuracy at baseline was high (93% on average), changed little over time, and was subject to ceiling effects; hence, only reaction time data were analysed further. Reaction time decreased linearly with repeated exposures to the test (main effect of time-point, *P < *0.0001). The Therapy ANOVA showed a trend towards a Block × Word list interaction driven by greater improvements for trained words (*P* = 0.050). There was also a Block × tDCS Group interaction (*P < *0.05), but it was driven by greater improvements with sham than with tDCS.

To assess if reading for meaning improved to a greater extent for those with impairments in the semantic domain at baseline, changes in reaction time over Block 1 (T4–T3) and Block 2 were compared to baseline scores on the Pyramid and Palm Trees Test. This revealed a significant positive correlation in both Block 1 (*r* = 0.7, *P* < 0.001) and Block 2 (*r* = 0.5, *P* < 0.05).

#### Sentence reading

Patients P7, P9 and P16 were unable to complete the Sentence Reading task: data are reported from 18 participants.

Picture verification accuracy at Baseline was high (87% on average), changed little over time, and was at ceiling in some participants. Only sentence reading speed in words per minute (wpm) was analysed further.

Average reading speed did not show a test-retest effect between Baseline and T3, but improved linearly during training (T3 to T5) and at the follow-up test (T6). The Therapy ANOVA showed an interaction between Word list and tDCS Group (*P < *0.05), but this interaction did not reflect a tDCS advantage: Group 1 participants improved more on words trained in Block 2 whereas Group 2 participants improved more on words trained in Block 1. As these improvements were consistent across Blocks 1 and 2 they could not be ascribed to tDCS stimulation, but instead reflected a difference between Group 1 and 2 participants.

#### Text reading

Patient P18 was unable to complete the Text Reading task. In the remaining 20 participants, there was little change over time in text reading accuracy, speed or comprehension. Neither the Omnibus MANOVA nor the Therapy ANOVAs identified any significant effects or interactions.

#### Sustained attention to response task

Due to a software malfunction, SART data were unavailable for Patient P9 at T5. Results are reported from the remaining 20 participants.

Small changes were observed between Baseline and T6: reaction times increased, false negative responses increased and false positives decreased, suggesting that participants responded more cautiously with repeated exposures to the test. However, the effect of time point was not significant in the Omnibus MANOVA, nor were any significant effects observed in the Therapy ANOVAs.

#### Self-report measures

The CDP was completed at T3 and T5 in 20 of 21 patients: Patient P4 declined to complete the questionnaire at T5. Ten of 20 patients reported improved reading ability (Fig. 6), but a Wilcoxon Signed Rank test showed this change was not significant (*T* = 98, *P* = 0.119).

Considering the four reading levels of the CDP separately, average improvements were largest for words (+0.43) and sentences (+0.35), but neither of these changes reached significance (*P* = 0.065 and *P* = 0.115, respectively).

When asked in the exit questionnaire whether participants thought their word reading had improved, 11/21 responded ‘a lot’; 9/21 responded ‘a little’; and only one responded ‘no’ (Patient P6). Nineteen of 21 participants said that they would like to continue using iReadMore (Patient P3 said ‘maybe’ and Patient P21 said ‘no’).

## Discussion

This study tested the efficacy of two concurrent therapies for central alexia: (i) iReadMore, a crossmodal, lexical word reading therapy; and (ii) anodal tDCS delivered to LIFG.

iReadMore improved word reading accuracy and reaction time for trained items, and, consistent with previous lexical therapies (Friedman and Robinson, 1991; Friedman *et al.*, 2002; Ska *et al.*, 2003; Kurland *et al.*, 2008; Lott *et al.*, 2008), did not generalize to untrained items. The unstandardized size of iReadMore’s effect on reading accuracy was 8.7% (95% CI 6.0–11.4) and the standardized effect size (Cohen’s *d*) was 1.38 (large). The effect size for reading reaction time was 100 ms (95% CI 56–145, *d* = 0.98, large).

Pretreatment reading of the high frequency, low imageability ‘core’ words was initially poor, but as a result of iReadMore training accuracy improved by 6% (95% CI 2.7 to 9.2, *d* = 0.78, moderate) and reaction time improved by 210 ms (95% CI 116–304, *d* = 1.00, large). The fact that these core words improved, coupled with the lack of evidence for an influence of word imageability or regularity on the therapy effects, suggests that the therapy can be effective for all word types.

Anodal tDCS paired with iReadMore had a small but significant facilitatory effect on word reading accuracy (2.6% on average, 95% CI − 0.1 to 5.3, Cohen’s *d* = 0.41), which generalized to untrained words. Anodal tDCS effects were not observed on word reading reaction time or on core word reading (accuracy or reaction time). This may be due to a lack of power to detect this small to medium effect size on a set of only 50 core words; or it may be because the same core words were trained twice, once with anodal tDCS and once with sham tDCS, meaning that the comparison between real and sham blocks was confounded by carry over effects from the preceding block.

In real-word terms, two blocks of iReadMore and anodal tDCS therapy (70 h training and 11 stimulation sessions in total) on all 350 trained words (two blocks of 150 words plus 50 core words), patients on average could read 29 more words, with a range based on the 95% CIs from 19 to 39 words. Patients were also on average 116 ms faster per trained word (ranging from 65 to 168 ms).

Therapy effects on reading accuracy (but not reaction time) remained significantly above baseline levels at the T6 follow-up session, 3 months after cessation of training. For core words, both accuracy and reaction time gains were maintained. However, the diminution of the effect size at T6 suggests that a maintenance dose of training may be required to keep up the benefits gained from the therapy.

The iReadMore therapy was designed to strengthen connections between orthographic (O), phonological (P) and semantic (S) representations. While improved oral word reading indicated improved access from orthography to phonology, improvement on the semantic matching task would have demonstrated strengthening of connections with semantic representations. In fact, the effect of iReadMore on semantic matching was very close to significance (*P* = 0.050). This result may have been subject to ceiling effects, as seven patients were within the control reaction time range on this task; hence we speculate that iReadMore may benefit reading for meaning in patients who have deficits in the semantic domain. This impression is supported by the positive correlation between greater semantic impairment (as measured by the Pyramids and Palm Tree Test at baseline) demonstrated greater improvements in semantic matching reaction time.

Training effects were observed at the word level, and did not generalize to sentence or text reading. This indicates that further text training (e.g. Multiple Oral Reading, Moyer, 1979; or Oral Reading for Language in Aphasia, Cherney, 2004) or multi-level training (Brown *et al.*, 2015) may be required to overcome the additional syntactic, semantic or verbal working memory deficits that impede text reading in central alexia.

The hypothesis that anodal tDCS delivered to LIFG would facilitate iReadMore training was also borne out. Compared to sham, anodal tDCS increased gains in reading accuracy for both trained and untrained words. There are at least two possible mechanisms of this improvement. The LIFG and adjacent premotor cortex are known to play an early, automatic role in phonological processing during reading (Cornelissen *et al.*, 2009; Wheat *et al.*, 2010; Woodhead *et al.*, 2014; Hoffman *et al.*, 2015). An effective connectivity study showed feedback connections from the LIFG to visual cortex were strengthened by reading training (Woodhead *et al.*, 2013); hence it is plausible that LIFG stimulation may enhance feedback and facilitate therapy effects, either by improving the veracity of the phonological representations themselves, or improving mappings between orthography and phonology via strengthened prediction error. The observation that anodal stimulation facilitated oral reading accuracy but not written semantic matching supports the inference that anodal tDCS delivered to LIFG acted upon phonological rather than semantic representations.

Alternatively, anodal tDCS may have enhanced the LIFG’s role in speech production (Hickok and Poeppel, 2007), consistent with anodal tDCS effects observed in anomic aphasia (Baker *et al.*, 2010; Marangolo *et al.*, 2011, 2013; Campana *et al.*, 2015). This would explain the generalization of our anodal tDCS effects to untrained words, but would predict improved speech output in the text reading task, which was not observed. An anodal tDCS induced increase in arousal or attention giving rise to these results is unlikely as we saw no effect on the patients’ performance in a test of sustained attention, the SART. This also suggests that the positive behavioural results of our study cannot simply be explained by non-specific excitation of the entire brain.

As an emerging clinical research tool, anodal tDCS has a number of outstanding questions about its mechanisms of action and the anatomical specificity of the stimulation effects (Schlaug *et al.*, 2008; Stagg and Nitsche, 2011; Parkin *et al.*, 2015; Fertonani and Miniussi, 2017). Finite modelling studies have suggested that distant bipole montages, such as used here, result in a wide spread of stimulation across the frontal lobe (Datta *et al.*, 2013). Other reports stress the importance of the interaction between stimulation and the underlying neural network activity especially for cognitive/language functions (Fertonani and Miniussi, 2017). In this context the overall effect of tDCS depends on the excitability of the stimulated brain area, meaning that even if the spread of electrical current is large, it will only serve to facilitate functionally engaged brain regions that are co-activated by the task being performed.

A previous reading training study of alexic patients showed that therapy strengthened LIFG feedback to visual cortex (Woodhead *et al.*, 2013). Importantly the LIFG was anatomically intact for all patients in this study; hence it is plausible that anodal tDCS delivered to LIFG may have facilitated iReadMore therapy effects either by direct enhancement of LIFG activation itself or by modulation of LIFG connectivity within the patients’ task engaged residual reading network.

Moreover, we demonstrated for the first time that repeated anodal tDCS sessions not only resulted in enhanced improvement for specifically trained reading materials but also in enhanced transfer effects to untrained reading materials. Our findings are thus in line with data from animal models (Fritsch *et al.*, 2010) healthy individuals (Reis *et al.*, 2009, 2015) and anomic stroke patients (Vestito *et al.*, 2014; Meinzer *et al.*, 2016) suggesting that multisession tDCS improves memory consolidation by impacting on plasticity-related protein synthesis, which is thought to be enhanced by concurrent application of tDCS during training. Magnetoencephalography data were acquired for these patients during the iReadMore trial, and we will use these to investigate the neural network changes that supported the behavioural changes reported here in a future study.

Whilst we set out to test the effects of iReadMore and anodal tDCS for patients with any type of central alexia, all but one participant (Patient P4) had phonological or deep alexia; hence, the applicability of these findings to surface alexia is limited. However, Patient P4’s results were consistent with the group average, suggesting that the therapy may benefit phonological and surface alexia alike. A post-release trial of the iReadMore app (http://www.ucl.ac.uk/aphasialab/apps/ireadmore.html) will aim to test a larger sample of patients in order to assess its efficacy for surface, deep, phonological and also pure alexia. The iReadMore app is available to the public.

## Funding

This trial was supported by the Medical Research Council (MR/K022563/1). J.C. is supported by a Wellcome Trust Senior Research Fellowship in Clinical Science (106161/Z/14/Z).

## Supplementary material

Supplementary material is available at *Brain* online.

## Supplementary Material

Supplementary Data

## Abbreviations

CAT: Comprehensive Aphasia Test
CDP: Communication Disability Profile
LIFG: left inferior frontal gyrus
O-P-S: orthographic-phonological-semantic system
tDCS: transcranial direct current stimulation
