# Supplementary material for: Randomized trial of iReadMore word reading training and brain stimulation in central alexia
Source: Brain. 2018 Jun 15;141(7):2127–41. doi: 10.1093/brain/awy138 (PMC6118228; doi:10.1093/brain/awy138)

Supplementary Table 1s.

Mean scores on all outcome measures at all time-points for tDCS Groups 1 and 2. G1=Group1, G2=Group2, Acc=accuracy, RT=reaction time, wpm=words per minute, CDP=Communication Disability Profile, B=Baseline.

**Measure**	**N**	**tDCS Crossover Group 1**					**tDCS Crossover Group 2**					**Omnibus (M)ANOVA**	**Therapy ANOVA**
	**G1:G2**	**B**	**T3**	**T4**	**T5**	**T6**	**B**	**T3**	**T4**	**T5**	**T6**	**(Baseline to T6)**	**(Block1 and Block2)**
**Word Reading, Acc (%)**	10 : 11											Time-Point: F=16.6,	Block: F=7.3, p<.05
Trained in Block1		54.2	56.6	67.1	63.3	61.6	56.5	59.7	67.8	65.3	65.4	p<.0001	Word-List: F=10.1, p<.005
Trained in Block2		54.4	56.6	60.6	66.8	61.0	56.1	58.1	58.3	68.2	63.2		**Block x Word-List: F=23.3, p<.0005**
Untrained		54.0	57.9	58.4	59.9	59.1	56.0	57.9	58.8	59.9	60.4	Word-List: F=5.7, p<.01	**Block x tDCS: F=5.3, p<.05**
**Word Reading, RT (ms)**	10 : 10												**Block x Word-List: F=7.1, p<.05**
Trained in Block1		1291	1228	1126	1208	1199	1122	1175	1021	1047	1085	**Time-Point x Word-List:**	
Trained in Block2		1339	1220	1214	1087	1261	1124	1192	1073	1054	1067	**F=9.3, p<.05**	
Untrained		1380	1282	1206	1202	1220	1107	1155	1047	1043	1097		
**Core Word Reading, Acc (%)**	10 : 11	48.2	54.0	58.1	58.0	56.5	42.9	43.9	51.1	51.7	51.7	Time-Point: F=20.1,	Time-Point: F=7.0, p<.005
**Core Word Reading, RT (ms)**	9 : 9	1419	1329	1372	1090	1168	1096	1162	1004	990	1014	p<.0001	Time-Point: F=4.4, p<.05
**Semantic Matching, Acc (%)**	9 : 10											Not analysed due to ceiling	Not analysed due to ceiling effects
Trained in Block1		94.4	90.7	93.1	91.7	93.5	92.5	95.4	93.8	93.3	92.9	effects	
Trained in Block2		94.4	90.3	92.6	94.0	89.8	91.3	93.3	93.8	93.3	93.3		
Untrained		94.0	89.4	89.4	90.7	90.3	92.1	95.0	90.4	91.7	92.5		
**Semantic Matching, RT (ms)**	9 : 10											Time-Point: F=7.0, p<.05	Block: F=5.1, p<.05
Trained in Block1		5065	3419	3075	2905	3299	4945	4023	3197	3424	3333		**Block x Word-List: F=4.4, p=.05**
Trained in Block2		5350	3457	3332	2897	3170	4813	4244	3533	3538	3757		Block x tDCS: F=6.9, p<.05
Untrained		5094	3366	3327	2880	3201	4972	4625	4031	3880	4000		
**Sentence Reading, Acc (%)**	8 : 10											Not analysed due to ceiling	Not analysed due to ceiling effects
Trained in Block1		87.5	90.0	91.3	91.3	93.8	84.0	86.0	87.0	85.0	87.0	effects	
Trained in Block2		87.5	86.3	88.8	85.0	87.5	91.0	87.0	88.0	91.0	94.0		
Untrained		83.8	83.8	86.3	82.5	82.5	88.0	88.0	91.0	86.0	95.0		
**Sentence Reading, Speed (wpm)**	8 : 10											Time-Point: F=3.7, p<.05	Word-List x tDCS: F=6.6, p<.05
Trained in Block1		79.9	85.4	92.1	91.1	104.9	80.0	72.0	96.0	104.9	96.8		
Trained in Block2		78.7	77.5	92.7	94.2	110.4	76.0	74.6	93.1	96.3	87.3		
Untrained		76.7	80.9	90.8	92.0	117.8	77.3	78.1	93.0	96.6	96.9		
**Text Reading**	9 : 11											Not significant	Not significant
Accuracy (%)		70.7	69.2	72.8	71.4	69.3	66.7	64.8	37.2	66.2	68.9		
Speed (wpm)		32.5	32.0	35.0	34.3	36.0	25.5	26.5	28.0	27.7	26.0		
Comprehension (/12)		5.8	7.0	7.4	6.3	6.2	6.3	5.6	7.5	7.6	7.2		
**SART**	10 : 11											Not significant	Not significant
False Positives (/24)		9.8	8.3	8.5	8	8.2	6.7	7.0	6.6	3.6	4.7		
False Negatives (/192)		6.6	3.9	5.6	6.6	7.8	4.3	8.5	6.7	6.5	8.4		
Go Trial RT (ms)		495	488	472	459	506	367	398	400	405	405		
**CDP**	10 : 10											Not applicable	Not applicable
Single Words (/4)		-	2.50	-	2.85	-	-	2.30	-	2.80	-		
Sentences (/4)		-	2.20	-	2.65	-	-	2.00	-	2.25	-		
Text (/4)		-	1.30	-	1.50	-	-	1.00	-	1.30	-		
Letters (/4)		-	1.40	-	1.10	-	-	1.00	-	1.25	-		

Supplementary Methods

Sample Size Calculation

A previous study using a prototype of iReadMore in a group of stroke patients with chronic pure alexia (Woodhead et al., 2013) resulted in an improvement in word reading reaction times of 149.0ms (sd = 214.5ms). This effect size was the change in trained word reading reaction times before (T2) minus after (T3) training. The sample size required to detect a comparable improvement was calculated using an online calculator from https://www.dssresearch.com/KnowledgeCenter/toolkitcalculators/samplesizecalculators.aspx. Using alpha = 5% and beta = 10%, a required sample size of n = 18 was indicated.

The expected effect size resulting from A-tDCS to the left IFG was powered based on a study by Baker and colleagues (2010). This study compared a-tDCS and sham during anomia therapy in 10 patients with chronic aphasia. On average, they observed a 14.4% improvement in picture naming accuracy following a-TDCS, compared to only 6% following sham. The benefit of a-tDCS over sham was 8.4% (s.d. = 10.2). The sample size required to detect a comparable improvement was calculated using the same calculator, again with alpha = 5% and beta = 90%, which indicated a sample size of n=13 would be required.

Training and Testing Word Lists

This word selection process is depicted below in Supplementary Figure 1s.

Baseline Testing (T1/T2): All participants were tested with all 590 words at baseline (split over T1/T2 sessions). The A, B and C lists comprised 180 words each and were matched for psycholinguistic variables. A list of 50 ‘Core’ words with very high written frequency was also included.

Training Word Lists: After T2, a subset of words (150 word triplets) was selected for use in the training. One list was used for Block1, one for Block2, and one was left untrained. The selected items from the A, B and C lists were different for each participant but were matched within-subject for psycholinguistic variables and baseline performance (word reading accuaracy and RT). All 50 Core words were trained in both Block1 and Block2.

Testing Word Lists: A further subset of words (90 word triplets) was selected for use in all subsequent word reading tests (T3-T6). The selected items from the A, B and C lists were different for each participant, but were matched within-subject for psycholinguistic variables and baseline performance. A subset of 30 Core words was also selected for testing at T3-T6.

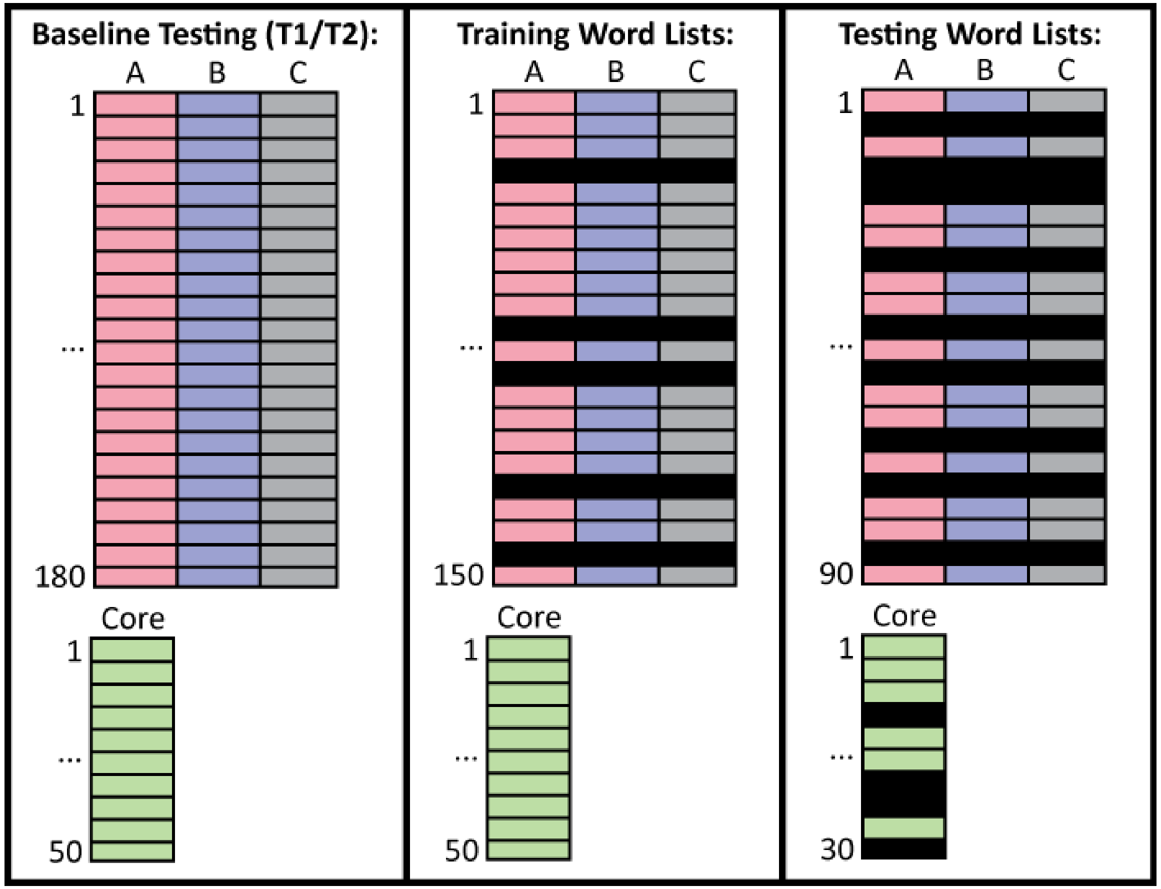

Supplementary Figure 1s

590 words (180 A, B and C words, plus 50 Core words) were tested at Baseline (across T1 and T2 sessions); from these, subject-specific word lists were selected for training (150 A, B and C words, plus 50 Core words); and from these, subject-specific word lists were selected for testing (90 A, B and C words, and a set list of 30 Core words).

iReadMore Training

<u>Training Words and Pictures</u>

The full training corpus of possible target words consisted of a picture representing the word, and a set of up to 9 paired written words to use as easy, medium or hard distractor items for each target word.

'Easy' distractor words shared only the first letter in common with the target word. 'Medium' distractor words shared at least 2 letters in common. 'Hard' distractor words (for words > 3 letters only) shared more than 2 letters in common.

The pictures were colour photos or drawings representing the target word. The representations of low imageability target words were abstract – see examples below. Even if these representations were not immediately understood, they became learnt through repeated exposure to allow pictorial priming of written word recognition.

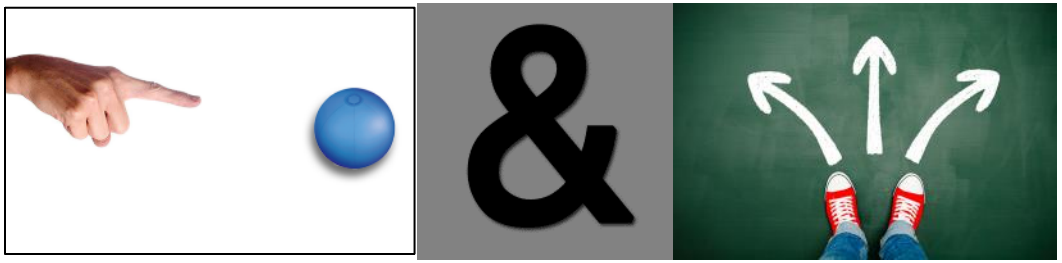

Examples of low imageability pictures: 'that', 'and', and 'any'."

A customised list of all 200 words to-be-trained (150 words selected from list A, B or C, plus all 50 Core words) was created for each subject in each block. The iReadMore software started with a list of these words in a randomized order.

<u>Exposure Phase</u>

In the first exposure phase, the first 10 items from the list were selected. The order of the word list adjusted in response to the participant's performance in the challenge phase. The 10 words at the top of the list were selected for each subsequent exposure phase.

Each exposure phase comprised 10 trials. Initiation of each trial was self-paced. In each trial, the picture representing the target word was presented, followed by simultaneous presentations of the written and spoken word-forms. Written word duration initially matched the patient's baseline word reading speed, then adapted according to performance in the subsequent challenge phase (see <u>Difficulty Adaptation: Global Parameters</u> below). Stimuli recordings from a female or a male speaker were randomly selected for each trial.

Challenge Phase

Challenge phases comprised up to 30 trials (3 repetitions of the 10 target words presented in the preceding exposure phase), but ended when the criterion score was reached. The criterion score adapted according to performance (see Difficulty Adaptation: Global Parameters).

In each trial, a spoken word from the preceding exposure phase was presented with a written word. The written word duration initially matched the patient's baseline word reading speed, then adapted according to performance (see Difficulty Adaptation: Global Parameters). In half the trials the written and spoken stimuli were the same word, and in half they were different. The selection of the distractor item in 'different' trials adapted according to performance on a word-by-word basis (see Difficulty Adaptation: Item-Specific Parameters). Participants made a same/different response via button press and received immediate feedback. Two points were awarded for a fast correct response; one for a slow correct response; and minus one for an incorrect response. The criterion duration for fast and slow responses adapted according to performance (see Difficulty Adaptation: Global Parameters). If the participant reached the criterion score within 30 trials, they passed that level and task difficulty increased in the next exposure phase.

Difficulty Adaptation: Global Parameters

Task difficulty was reflected in three global adaptive parameters: 1) written word duration in exposure and challenge phases; 2) criterion score in the challenge phase; and 3) criterion reaction times for fast/slow correct responses in the challenge phase. All three parameters changed simultaneously when the difficulty level changed. The difficulty level began at 1, then increased incrementally when the participant passed a challenge phase. If the participant failed three successive challenge phases, then the level decreased by one. The formulae for generating the task parameters at each difficulty level ('LEVEL') are shown in the table below. The word duration was initially set to the participant's average word reading RT at baseline ('baseline_RT'). Each parameter had a maximum or minimum boundary that could not be exceeded – once this was reached, the parameter remained constant, but could revert to an easier setting if the difficulty level subsequently reduced.

**Parameter (y)**	**Function**	**Min / max allowed**
Word duration (ms)	y = baseline_RT – 2 * LEVEL * baseline_RT / 100	Min = 100
Criterion score	y = 20 + 0.5 * LEVEL	Max = 56
Fast response criterion (ms)	y = 4000 – 30 * LEVEL	Min = 2000
Slow response criterion (ms)	y = 10000 – 90 * LEVEL	Min = 5000

Difficulty Adaptation: Item-Specific Parameters

Task difficulty was also reflected in two item-specific adaptive parameters: 1) the distractor difficulty level in the same/different task (easy, medium or hard), and 2) the position of the target word in the word list, which affected how soon the word would appear again in a subsequent exposure phase.

The distractor word selected for each 'different' trial in the challenge phase started at the easy level. In each challenge phase, a target word could be presented up to three times, and 0-3 of those trials could be 'different' trials. Distractor difficulty level (easy / medium / hard) in subsequent challenge phases was then adapted according to the following rules:

1) If a DIFFERENT trial appeared once, the distractor difficulty level moved forwards (+1) if the response was correct, or moved backwards (-1) if the response was incorrect).
2) If a DIFFERENT trial appeared more than once, the outcome for all trials was summed, e.g. +1 for each correct trial, and -1 for each incorrect trial. If the summed value was POSITIVE then the difficulty level moved upward, if it was NEGATIVE the difficulty level moved down; and if it was ZERO it stayed the same.

The position of the target word in the word list changed according to performance on all 'same' or 'different' trials for that word in the challenge phase. Each target word would be presented up to three times in each challenge phase. If any one of the trials was responded to incorrectly, the position of the target word in the word list would not change, so that the word would definitely appear in the next exposure phase. If all trials were responded correctly, the change in word position would be calculated by taking the average position change score using the following rules:

**Same / different trial**	**Easy / medium / hard level**	**Position change score**
Same	Easy	+ 20
Same	Medium	+ 20
Same	Hard	+ 20
Different	Easy	+ 20
Different	Medium	+ 50
Different	Hard	+200

The result of this position change score meant that words would be presented again soon (e.g. if the average position change score was low) or not for a long time (e.g. if the average position change score was high). The number of times that each target word in the word list was presented therefore depended on performance (see Participant Performance).

Participant Performance

Accuracy in the challenge phase was generally high, with participants answering 90.6% of trials correctly on average (s.d. = 8.4). Performance ranged from 65.0% to 97.4%. Data from all subjects can be seen in Supplementary Table 3s.

Due to the design of the item-specific difficulty adaptation, the number of times each word was presented during training correlated closely with accuracy for that word. On average over all participants and both blocks there were 76.6 presentations of each word per block, but this could vary widely depending on performance.

Central Alexia Subtypes

Participants were classified into central alexia sub-types (Surface dyslexia, Phonological dyslexia and Deep dyslexia) using classification criteria described by Whitworth et al (2014):

- Surface dyslexia (Marshall & Newcombe, 1973) was defined according to the presence of a regularity effect (better reading of regular compared to irregular words) and relatively preserved pseudoword reading. Word reading errors in surface dyslexia include regularisation errors (SEW>"Sue") and visual errors (SUBTLE>"Sublet").
- Phonological dyslexia was defined according to the presence of a lexicality effect (better word reading than pseudoword reading) and an imageability effect (better reading of high than low imageability words). Word reading errors include visual and/or semantic errors.
- Deep dyslexia was defined according to the presence of lexicality and imageability effects. Word reading errors include purely semantic errors as well as visual and/or semantic errors.

Word reading errors on the full corpus of words tested at baseline were coded as phonological (including purely phonological errors, SEW→'sue'; and visual and/or phonological errors, DOOR→ 'doom'); semantic errors, (including purely semantic errors, APE→'monkey' and visual and/or semantic errors, CLING→'clasp'); or 'other' errors (including morphological errors, LOVELY →'loving'; and unrelated errors).

Regularity, imageability and length effects on word reading ability were identified using binary logistic regression on each participant's baseline word reading accuracy data. Only regression models that were significant were used for classification purposes: for some participants (P10, P11, P12 and P14) accuracy was very high and there was insufficient variance in the dependent variable to produce a significant model overall. In these cases, a linear regression analysis on word reading RT data was attempted, but none were significant.

Finally, the lexicality effect was determined by comparing overall percentage accuracy on the baseline word and pseudoword reading tests.

Supplementary Table 2s

ID	Word Reading Errors (%)			CAT Noun Naming Errors (%)			Whitworth Classification					CA Type
	Phonological	Semantic	Other	Phonological	Semantic	Other	Lexicality	Regularity	Imageability	WRT Semantic errors	Length	
**P1**	21.8	38.5	39.8	0.0	0.0	0.0	Y	N	Y	Y	N	D
**P2**	26.6	21.8	51.7	20.0	80.0	0.0	Y	N	Y	Y	Y	D
**P3**	35.6	8.1	56.4	100.0	0.0	0.0	Y	N	Y	N	Y	P
**P4**	89.7	2.2	8.0	25.0	37.5	37.5	N	Y	Y	N	Y	S
**P5**	53.1	9.4	37.5	0.0	100.0	0.0	Y	N	Y	N	N	P
**P6**	71.7	8.7	19.6	0.0	0.0	0.0	Y	N	Y	N	N	P
**P7**	62.6	3.1	34.4	0.0	71.4	28.6	N	N	Y	N	Y	D
**P8**	39.6	19.5	40.9	0.0	0.0	0.0	Y	N	Y	Y	Y	D
**P9**	39.5	11.1	49.4	25.0	75.0	0.0	N	N	Y	Y	Y	D
**P10**	94.7	0.0	5.3	0.0	0.0	100.0	Y	*	*	N	*	P
**P11**	72.1	2.3	25.6	100.0	0.0	0.0	Y	*	*	N	*	P
**P12**	100.0	0.0	0.0	0.0	0.0	100.0	Y	*	*	N	*	P
**P13**	66.2	7.4	26.5	60.0	20.0	20.0	Y	N	N	N	Y	P
**P14**	64.5	9.0	26.5	60.0	30.0	10.0	Y	*	*	N	*	P
**P15**	6.4	76.6	17.0	0.0	75.0	25.0	Y	N	Y	Y	Y	D
**P16**	50.4	8.1	41.5	66.7	16.7	16.7	Y	N	N	N	Y	D
**P17**	60.3	1.6	38.1	78.6	0.0	21.4	Y	N	Y	N	Y	P
**P18**	31.8	10.3	57.9	40.0	20.0	40.0	N	Y	Y	Y	Y	D
**P19**	46.4	10.6	43.0	0.0	100.0	0.0	Y	N	Y	N	N	P
**P20**	75.7	2.1	22.2	75.0	0.0	25.0	Y	N	Y	N	Y	P
**P21**	17.0	33.9	49.2	100.0	0.0	0.0	Y	N	Y	Y	N	D

*Regression analysis was used to identify the subtype patterns. The accuracy score for these participants were too high to perform the analysis and regression analysis of reaction time data was uninformative.

[1] Patients were classified into Central Alexia subtype according to the criteria outlined in Whitworth, Webster, & Howard (2014)

[2] Naming errors were taken from the CAT naming nouns test. In order to replicate the analysis of word reading, non-responses were excluded from the analysis.

Supplementary Table 3s

ID	iReadMore Training Dose		iReadMore Training Data	
	Block1	Block2	% Accuracy	# Presentations
**P1**	35.33	35.03	97.1	77.8
**P2**	37.35	40.02	90.0	80.7
**P3**	36.00	36.07	95.8	86.0
**P4**	35.10	41.37	83.8	71.9
**P5**	35.07	32.00	96.1	81.6
**P6**	35.15	36.02	95.2	81.3
**P7**	35.42	37.68	77.6	74.5
**P8**	35.23	38.05	95.6	80.2
**P9**	35.52	35.25	65.0	72.5
**P10**	35.60	35.88	95.7	74.3
**P11**	35.90	35.17	97.1	88.3
**P12**	35.13	35.18	97.2	82.6
**P13**	32.15	33.05	93.9	66.2
**P14**	35.03	34.95	92.7	74.1
**P15**	36.03	36.02	91.8	74.0
**P16**	30.05	32.72	90.0	65.2
**P17**	35.00	35.00	97.4	82.5
**P18**	29.85	30.25	88.4	65.8
**P19**	32.57	34.33	79.9	81.8
**P20**	36.05	35.52	84.1	75.0
**P21**	32.18	30.05	97.2	72.9

Supplementary Figure 3s. Subject specific therapy effects on word reading accuracy

Subject specific therapy effect on single word reading accuracy for i) words Trained in Block1 (blue), ii) words Trained in Block2 (red), iii) Untrained words (black). Training Block1 was administered between T3 and T4 (shaded blue); Block2 was administered between T4 and T5 (shaded pink). tDCS Group1 received Anodal tDCS (A-tDCS) over Block1 and sham tDCS (S-tDCS) over Block2, whereas tDCS Group2 received the stimulation types in the reverse order. Cross-hatching indicates the A-tDCS block on each plot. Graphs use either a 40-percentage point or 20-percentage point scale, depending on the individual's performance.

tDCS Group 1 (Block1: A-tDCS; Block2: Sham)

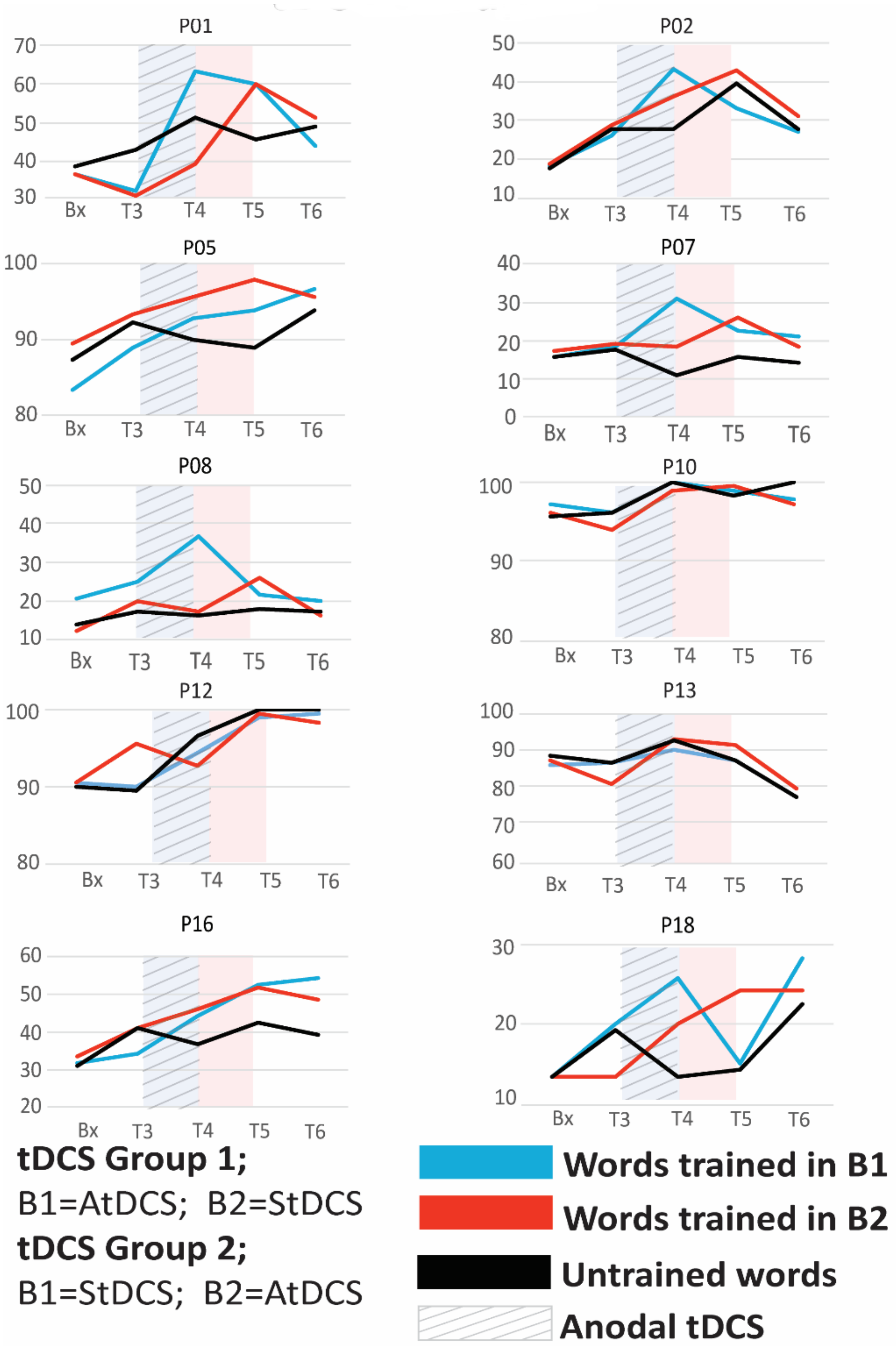

tDCS Group 2 (Block1: Sham; Block2: A-tDCS)

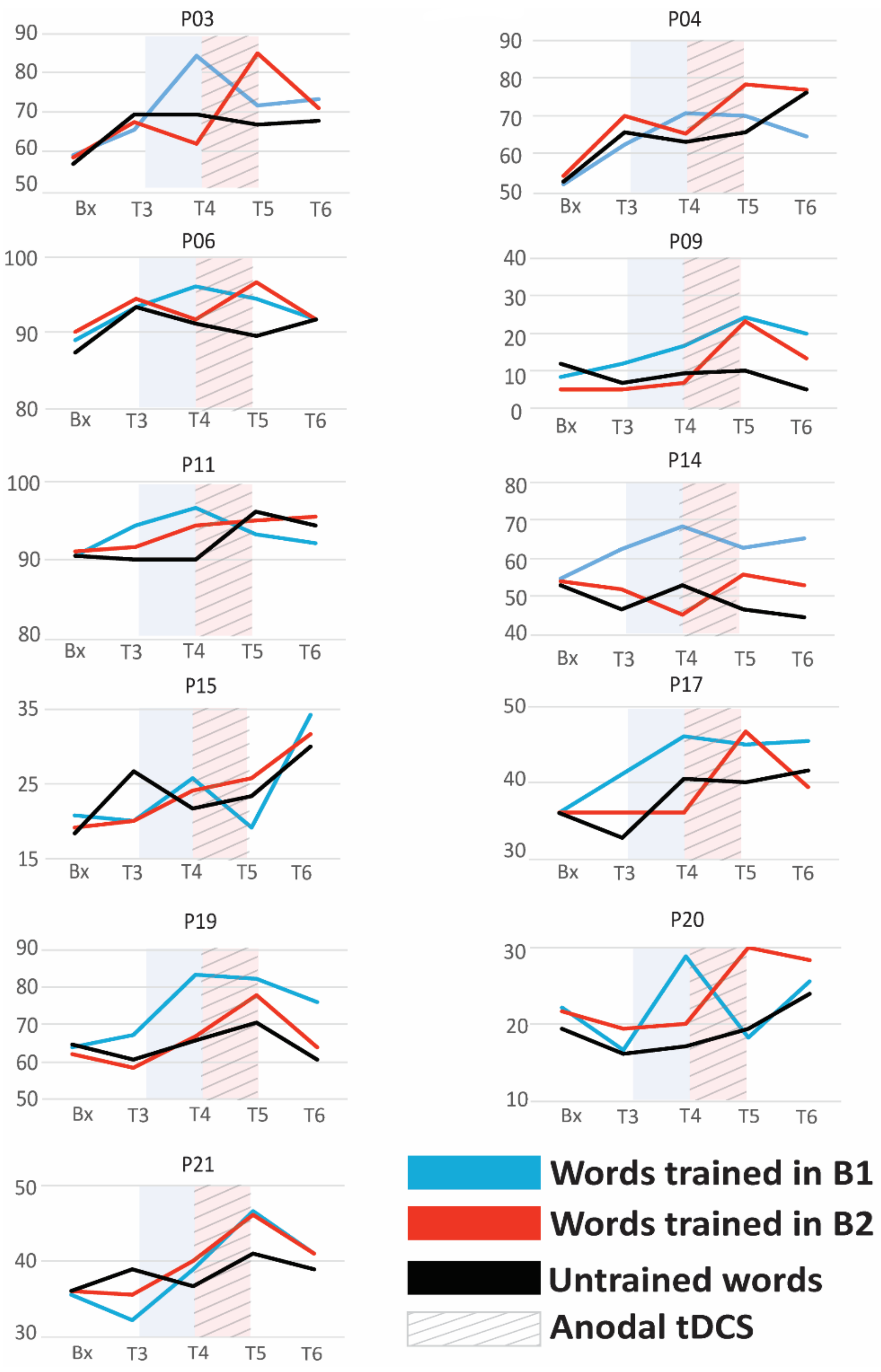

Supplement: Supplementary Data [file awy138_Suppl_data.pdf]
